# The Effect of Pylorus Removal on Delayed Gastric Emptying after Pancreaticoduodenectomy: A Meta-Analysis of 2,599 Patients

**DOI:** 10.1371/journal.pone.0108380

**Published:** 2014-10-01

**Authors:** Wenming Wu, Xiafei Hong, Lilan Fu, Shanglong Liu, Lei You, Li Zhou, Yupei Zhao

**Affiliations:** Department of General Surgery, Peking Union Medical College Hospital, Chinese Academy of Medical Sciences and Peking Union Medical College, Beijing, China; The First Affiliated Hospital of Nanjing Medical University, China

## Abstract

**Background:**

Delayed gastric emptying is a serious complication of pancreaticoduodenectomy. The effect of pylorus removal on delayed gastric emptying has not been well evaluated.

**Study Design:**

We searched five databases (PubMed, EMBASE and the Cochrane Central Register of Controlled Trials, Scopus and Web of Science) up to July 2014. The meta-regression analysis was performed to evaluate any factors accountable for the heterogeneity. Publication bias was assessed by Egger's test, and corrected by Duval's trim and fill method. Subgroup analyses were conducted for different surgical techniques of pyloric removal. Other intraoperative and postoperative parameters were compared between two groups.

**Results:**

We included 27 studies involving 2,599 patients, with a moderate-high heterogeneity for primary outcome (*I^2^* = 63%). Meta-regression analysis showed that four variables primarily contributed to the heterogeneity, namely nasogastric tube intubation time, solid food start time, preoperative diabetes percentage and the number of patients in pylorus-preserving group. After excluding four studies, the remaining twenty-three studies showed reduced heterogeneity (*I^2^* = 51%). Then we used Duval's trim and fill method to correct publication bias. The corrected MH odds ratio was 0.78 (95% CI: 0.52–1.17). A subgroup analysis showed that pylorus removal tends to reduce delayed gastric emptying incidence for subtotal stomach-preserving pancreaticoduodenectomy or pylorus-resecting pancreaticoduodenectomy, compared with pylorus-preserving group. However, standard Whipple procedure failed to show any significant reduction of DGE compared with pylorus-removal group. No significant differences were observed in terms of length of hospital stay, infection and pancreatic fistula; however, pylorus removal resulted in longer operation time, more blood loss and higher mortality.

**Conclusion:**

The pylorus removal does not significantly reduce the overall incidence of delayed gastric emptying. Subtotal stomach-preserving pancreaticoduodenectomy or pylorus-resecting pancreaticoduodenectomy tends to reduce delayed gastric emptying incidence, but needs further validation.

## Introduction

Pancreaticoduodenectomy is an important surgical intervention for periampullary diseases. According to resection extent of pyloric region, pancreaticoduodenectomy uses either pylorus-preserving or pylorus-removing procedures. Specifically, pylorus-preserving pancreaticoduodenectomy (PPPD) preserves all of the stomach and part of the proximal duodenum [1], [2]. On the contrary, pylorus-removing pancreaticoduodenectomy (PRPD) includes different surgical techniques, namely the standard Whipple procedure (SWP), subtotal stomach-preserving pancreaticoduodenectomy (SSPPD) and pylorus-resecting pancreaticoduodenectomy (PrPD). SWP usually resects the pylorus along with 30–40% of the distal stomach [3]. SSPPD and PrPD involve the resection of the pyloric ring together with 2–3 cm of the distal stomach and preserve the majority of the stomach [3], [4].

Delayed gastric emptying (DGE) is a serious postoperative morbidity of pancreaticoduodenectomy. DGE may prolong hospital stay, compromise the quality of life, and impair long-term nutrition status. The International Study Group of Pancreatic Surgery (ISGPS) has proposed a grading system to catogorize different level of severity of DGE. ISGPS grade B/C DGE usually has more clinical impacts since it might change the postoperative clinical management. In additional, ISGPS grade C DGE are more frequently seen with other postoperative complications [5], [6].

It is believed that postoperative pyloric dysfunction will predispose a patient to DGE. Pancreaticoduodenectomy may cause pyloric dysfunction due to the devascularization and denervation of the pyloric region. Inadequate blood supply that leads to ischemic gastroparesis is one of the mechanisms that may cause DGE [7]. Pyloric denervation, such as vagal nerve injury, may cause pylorospasm and DGE [8]. The preservation of blood supply and innervation of the pyloric region or the dilatation of the pylorus may reduce the incidence of DGE [9], [10].

Previously meta-analyses [11]–[13] have compared the PPPD with SWP, and one recent meta-analysis analyzed the intraoperative and postoperative outcomes of several randomized controlled trials (RCTs) [14]. As for DGE, all of the meta-analyses indicated a high heterogeneity among studies. In fact, many preoperative and postoperative factors might influence incidence of DGE, including preoperative diabetes, pancreatic fistula, postoperative complications, preoperative biliary drainage, and method of reconstruction. These variables may account for the observed heterogeneity across individual studies [15]. In addition, the variations in the surgical techniques, specifically the proportion of the stomach that is resected during PRPD, may be a confounding variable; therefore, further subgroup analyses are needed.

In this study, we systematically evaluated the following questions: 1) Whether pyloric ring removal reduces the overall incidence of DGE; 2) The factors that contribute to the overall heterogeneity among studies; and 3) Whether the variations in the surgical techniques (PrPD/SSPPD versus SWP) lead to different results with regard to the incidence of DGE.

## Materials and Methods

### Review Strategy and Quality Assessment

The review process was adhered to the Preferred Reporting Items for Systematic Reviews and Meta-Analyses statement [16]. The literature search strategy and eligibility criteria for inclusion and exclusion of studies and study outcomes were specified in advance to avoid bias. We searched PubMed, EMBASE, the Cochrane Central Register of Controlled Trials (CENTRAL), Scopus and Web of Science for studies published up until July 2014, without restrictions placed on the language or publication date. The search strategy was as follows:

*PubMed*: ‘pancreaticoduodenectomy’ AND (‘pyloric’ OR ‘pylorus’) (in Title/Abstract)

*EMBASE*: ‘pancreaticoduodenectomy’ AND (‘pyloric’ OR ‘pylorus’) (in Keywords with ‘Map Term to Subject Heading’)

*CENTRAL* and *Scopus*: ‘pancreaticoduodenectomy’ AND (‘pyloric’ OR ‘pylorus’) (in Title, Abstract, Keywords)

*Web of Science*: ‘pancreaticoduodenectomy’ AND (‘pyloric’ OR ‘pylorus’) (in Topic)

All abstracts were retrieved and reviewed independently by two authors according to the eligibility criteria (listed below). A third author supervised the review process and settled disagreements on the study inclusion. To determine the inconsistency between reviewers, an inter-reviewer reliability analysis was conducted using a kappa statistic. If a study was included, the full-text of the article was retrieved and the relevant data were extracted. If additional data were considered to be necessary, the reviewers would contact the authors of the original articles. We evaluated the quality and the risk of bias of nonrandomized studies using the Newcastle-Ottawa quality assessment tool [17].

### Eligibility Criteria

Inclusion and exclusion criteria were pre-defined to avoid bias.

#### Inclusion criteria

(1) Studies had to feature a comparison between PRPD and PPPD; and (2) the sample size in each surgical procedure should be greater than 10 patients.

#### Exclusion criteria

(1) Only data from a single group of patients (either PRPD or PPPD) were reported; (2) the incidence of DGE was not reported; or (3) the article was published in the form of a case report, review article, letter to the editor, editorial or conference abstract.

### Outcomes

#### Primary outcomes

(1) Incidence of DGE

Definition: An International Study Group of Pancreatic Surgery (ISGPS) grade of DGE B/C was calculated for studies that followed the scoring system of the ISGPS [6]. For those studies that did not use the ISGPS scoring system, the overall incidence of DGE was considered.

#### Secondary outcomes

(1) Blood loss, (2) operation time, (3) length of hospital stay, (4) mortality, (5) pancreatic fistula, and (6) infection.

### Statistical Analysis

For the baseline characteristics, we applied the *Chi*-squared test for categorical variables and Student's t-test for continuous variables. The mean and standard deviation (S.D.) of the continuous variables were estimated using the median, range and the number of patients [18]. *P-values* less than 0.05 were considered statistically significant. With respect to the outcomes, data from the original articles were extracted and analyzed using the Review Manager 5.1 software (Cochrane Collaboration). The *I^2^* index was used as an indicator of the between-study heterogeneity. We used meta-regression to identify potential variables causing the heterogeneity. The meta-regression analysis and Duval's trim and fill correction were conducted by Comprehensive Meta Analysis Version 2.2 (Biostat, Englewood, NJ, USA). For meta-regression analyses, the factors were predefined. All of the following factors were previously reported to influence the incidence of DGE and were thus used as variables: preoperative diabetes, pancreatic fistula, postoperative complications, preoperative biliary drainage, method of reconstruction, percentage of malignancies, DGE evaluation method, nasogastric tube (NGT) intubation and solid food start time for the conventional method, and the use of prokinetic medicine [15], [19], [20]. Other common variables that were also routinely included were publication year, study design, total patient number, patient number in PRPD group, patient number in PPPD group, blood loss, operation time and infection. Subgroup analyses were conducted to assess the different surgical techniques of PRPD.

## Results

### Literature Search

**Figure 1:**
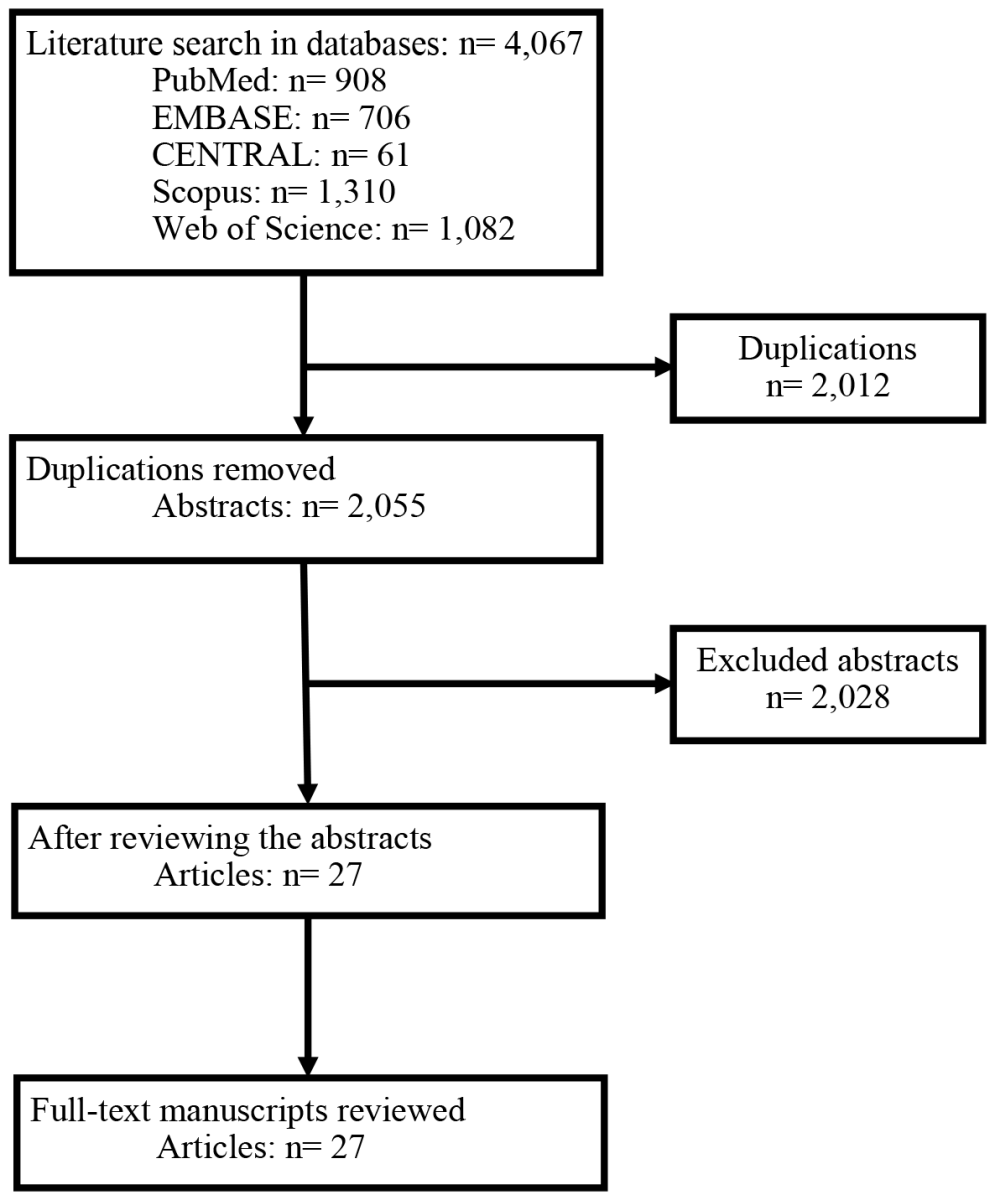
Flowchart of the literature search and studies inclusion. Abbreviations: CENTRAL, Cochrane Central Register of Controlled Trials.

Our search strategy yielded 4,076 abstracts from the aforementioned five databases. After the exclusion of duplications, two reviewers independently reviewed 2,055 abstracts. In this step, case reports, review articles, letters to the editor, editorials and conference abstracts were excluded. Abstracts that did not compare PRPD with PPPD or that did not report the incidence of DGE were also excluded. Thus, twenty-seven original articles were included, and the full-text of the manuscripts were read and evaluated [3], [4], [19], [21]–[44]. Finally, we included all twenty-seven studies for this systematic review and meta-analysis with an inter-reviewer reliability of kappa  = 0.978 (P<0.001) (Figure 1).

2,599 patients were included (1,289 who underwent PRPD and 1,310 who underwent PPPD). The publication year ranged from 1992 to 2014. Seven studies were RCTs, eight studies were prospective nonrandomized studies, and another twelve studies were retrospective ones. Nineteen studies featured SWP as a procedure for the pyloric removal group; five studies used SSPPD, and two studies used PrPD. One study [23] featured both SSPPD and SWP as PRPD. The definition of DGE varied across the studies; therefore, we listed the detailed information for each in Table 1. The gender and age of patients in the two groups were not significantly different.

**Table 1:** Clinical characteristics of the included studies.

First author	Publi-cation year	Study design	Surgical techniques of PRPD group	DGE definition	Gender (M: F)			Age (Mean ± S.D.)		
					PRPD	PPPD	P value	PRPD	PPPD	P value
Akizuki [4]	2008	PNR	SSPPD	NGT ≥ POD 10 or Solid food ≥ POD 14	18, 12	20, 14	0.56	65±10	66±13	0.56
Di Carlo [21]	1999	PNR	SWP	NGT> POD 10 or Vomiting> POD 10	24, 15	46, 28	0.95	61.1±8.8	62.0±8.3	0.59
Duffy [22]	2003	Retro	SWP	NGT ≥ POD 7 or Oral Intake ≥ POD 7	14, 9	20. 12	1.00	67.3±9.3	67.3±14.0	1.00
Fujii [23]	2012	Retro	SSPPD SWP	ISGPS Grade B/C	74, 51	19, 14	1.00	63.7±7.3	63.8±12.0	0.95
Gao [24]	2007	Retro	SWP	NGT ≥ POD 10 or Solid food ≥ POD 14	64, 40	23, 19	0.57	58.2±11.9	58.4±14.5	0.93
Hackert [25]	2013	PNR	PrPD	ISGPS Grade B/C	22, 18	22, 18	1.00	65.0±10.0	65.0±10.0	1.00
Hayashibe [26]	2007	Retro	SSPPD	NGT ≥ POD 10 or Regular diet ≥ POD 14	8, 13	4, 8	0.70	64.3±9.5	60.9±8.5	0.40
Hoem [27]	2012	PNR	SWP		22, 16	24, 18	1.00	69.5±9.8	66.0±12.3	0.17
Horstmann [28]	2004	PNR	SWP	NGT> POD 7 or Regular diets> POD 14				62	59	
Jimenez [29]	2000	Retro	SWP	Oral intake> POD 14	16, 17	20, 19	1.00	50.0±15.5	45.0±15.8	0.18
Kawai [3]	2011	RCT	PrPD	ISGPS Grade B/C	38, 28	33, 31	0.60	67±9	68±9	0.58
Kurahara [30]	2010	Retro	SSPPD	ISGPS Grade B/C	38, 26	26, 22	0.58	66.8	66.4	0.24
Lin [31]	2005	RCT	SWP	NGT ≥ POD 10	13, 6	10, 4	1.00	66.7±9.5	64.5±8.5	0.50
Makhija [32]	2002	PNR	SWP	NGT> POD 7						
Matsumoto [19]	2014	RCT	SSPPD	ISGPS Grade B/C	35, 15	29, 21	0.30	67±9	66±10	0.81
Mosca [33]	1997	Retro	SWP	NGT> POD 14						
Nanashima [34]	2013	Retro	SSPPD	ISGPS Grade B/C	15, 12	21, 7	0.22	66±12	68±8	0.56
Paquet [35]	1998	RCT	SWP							
Patel [36]	1995	Retro	SWP	Oral fluids ≥7 POD	28, 24	6,9	0.39	63.0±15.0	61.3±13.6	0.70
Pirro [37]	2002	Retro	SWP	NGT> POD 7						
Roder [38]	1992	PNR	SWP		32, 30	28, 20	0.56	60.0±12.5	64.0±9.5	0.07
Seiler [39]	2005	RCT	SWP	NGT> POD 5	33, 33	36, 28	0.29	65.0±13.2	64.8±14.3	0.30
Srinarmwong [40]	2008	RCT	SWP	NGT ≥ POD 10 or Regular diet ≥ POD 90	8, 5	10, 4	0.69	62.5±5.8	62.2±6.6	0.93
Takahashi [41]	1999	Retro	SWP							
Tani [42]	2009	Retro	SWP	NGT ≥ POD 10 or Regular diet ≥ POD 14	11, 6	18, 20	0.26	65.0±12.6	69.3±8.8	0.15
Tran [43]	2004	RCT	SWP	NGT ≥ POD 10 or Regular diet ≥ POD 14	50, 37	58, 25	0.11	62.0±8.5	64.0±5.8	0.27
Van Berge Henegouwen [44]	1997	PNR	SWP	NGT ≥ POD 10 or Regular diet ≥ POD 14	55, 45	45, 55	0.20	58.8±9.0	62.0±10.0	0.10

Abbreviations: PRPD, pylorus-removing pancreaticoduodenectomy; PrPD, pylorus-resecting pancreaticoduodenectomy; SSPPD, subtotal stomach-preserving pancreaticoduodenectomy; SWP, Standard Whipple procedure; PNR, Prospective nonrandomized study. Retro, Retrospective Study; RCT, Randomized Controlled Trial; NGT, Nasogastric tube; POD, Postoperative day.

For nonrandomized studies, a modified table that included the key components of the Newcastle-Ottawa quality assessment tool was employed to assess the quality and the risk of publication bias [17], [45]. The modified table focused on the representativeness, comparability, ascertainment of exposure and follow-up. The overall bias was also estimated at the end of the table (Table S1).

### Primary Outcomes

#### The overall DGE incidence of PRPD to PPPD was not significantly different

First, we analyzed the effect of pylorus removal for all included studies. The *I^2^* index was 63%, which indicated that heterogeneity was moderate to high. We used meta-regression to explore the potential variables that might account for the heterogeneity. We used method of movement as the calculation method and considered a variable to be significant if the *P* value in ‘Model’ section was less than 0.05 (Table 2). Four variables showed statistical significance for the meta-regression, which were patient number in PPPD group, presence of preoperative diabetes, NGT intubation time and solid food start time. For each variable, we aimed to identify studies that might contribute to the heterogeneity. Two studies [26], [34] featured a longer NGT intubation period and solid food start time. In detail, the NGT intubation time was over POD 11 and the solid food start time was over POD 15 for these two studies. One of them [34] also had a higher rate of preoperative diabetes, with 67.3% of total patients diagnosed of diabetes. We also sequentially removed studies with the smallest number of participants in PPPD group. Three studies [26], [31], [40] was removed, with PPPD patient number of 12, 14 and 14, respectively. Therefore, four studies [26], [31], [34], [40] were removed from further analysis with a reduced heterogeneity (*I^2^* = 51%).

**Table 2:** Meta-regression for variables that influence the incidence of DGE.

Variables	Model		Residual	
	(Qmodel)	P value	(Qresid)	P value
Study Design (Randomized Versus Nonrandomized)	0.011	0.912	28.964	0.266
Surgery Type (SWP Versus SSPPD/PrPD)	3.320	0.068	30.248	0.215
Method of Reconstruction (Antecolic versus Retrocolic)	1.100	0.294	9.571	0.386
DGE Evaluation Method (Whether using ISGPS Grading System)	3.583	0.058	29.820	0.231
Prokinetic Medicine Usage (Whether routinely used)*	NA	NA	NA	NA
Total Patients Number	2.674	0.102	27.974	0.309
Patients Number in PRPD group	0.176	0.675	28.359	0.292
Patients Number in PPPD group	5.534	0.019	28.956	0.266
Publication Year	0.655	0.419	29.573	0.241
Operation Time	2.353	0.125	18.219	0.197
Blood Loss	0.126	0.722	17.224	0.244
Percentage of Malignancies	0.068	0.793	27.095	0.300
Pancreatic Fistula	2.018	0.155	28.096	0.256
Infection	0.013	0.908	25.957	0.302
Postoperative Complications	0.084	0.771	26.731	0.268
Preoperative Diabetes	10.271	0.001	4.861	0.431
Preoperative Biliary Drainage	0.087	0.767	3.364	0.339
NGT Intubation Period	4.104	0.043	7.442	0.490
Solid Food Start Time for Conventional Methods†	3.864	0.049	8.302	0.504

*Only one study reported the routine use of prokinetic medicine, and thus the meta-regression for this variable could not be conducted.

† Solid food start days were obtained from the original publications or from the authors. If this parameter was not available, the mean days solid food in the PPPD (the conventional method) group was used.

Abbreviations: PRPD, pylorus-removing pancreaticoduodenectomy; PrPD, pylorus-resecting pancreaticoduodenectomy; SSPPD, subtotal stomach-preserving pancreaticoduodenectomy; SWP, Standard Whipple procedure; NGT, Nasogastric tube.

**Figure 2:**
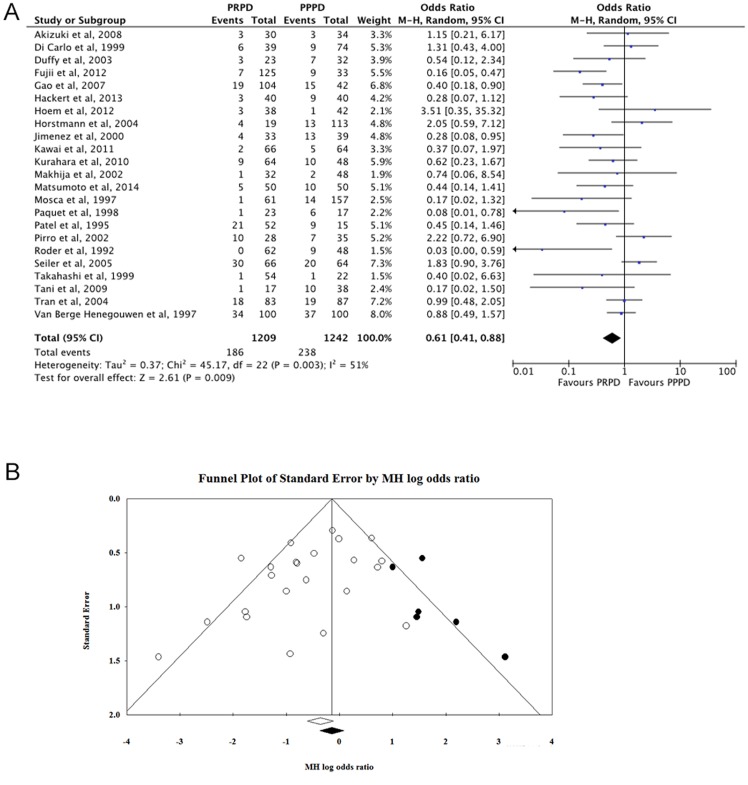
Delayed gastric emptying (DGE) incidence for overall studies. **A**. Meta-analysis of DGE of twenty-three studies. **B**. Funnel plot of Duval's trim and fill correction of DGE incidence for the twenty-three studies. Included studies are illustrated as white dots. Potential missing studies are illustrate as black dots.

The remaining 23 studies were included in the meta-analysis using a random-effect model. The MH odds ratio was 0.61 (95% CI: 0.41–0.88; *P*<0.01) (Figure 2A). However, publication bias was detected by Egger's test (*P* = 0.05). Then, Duval's trim and fill method was used to correct the result (Figure 2B). Six potential missing studies were replaced to the right of the mean odds ratio, which were illustrated as black dots. The MH odds ratio was 0.78 (95% CI: 0.52–1.17) after correction. Therefore, we concluded that the removal of the pylorus did not reduce the overall incidence of DGE.

Next, we wanted to evaluate whether different surgical techniques within the PRPD group would have different impacts on DGE. The PRPD group contained three different surgical techniques, namely SSPPD, PrPD and SWP. Two of them, SSPPD and PrPD, were similar, because both preserve the majority of the stomach. In contrast, SWP usually resects 20–40% of the stomach volume. Therefore, we conducted subgroup analyses of SWP, SSPPD, PrPD and SSPPD/PrPD, respectively.

#### The DGE incidence of SWP to PPPD was not significantly different

**Figure 3:**
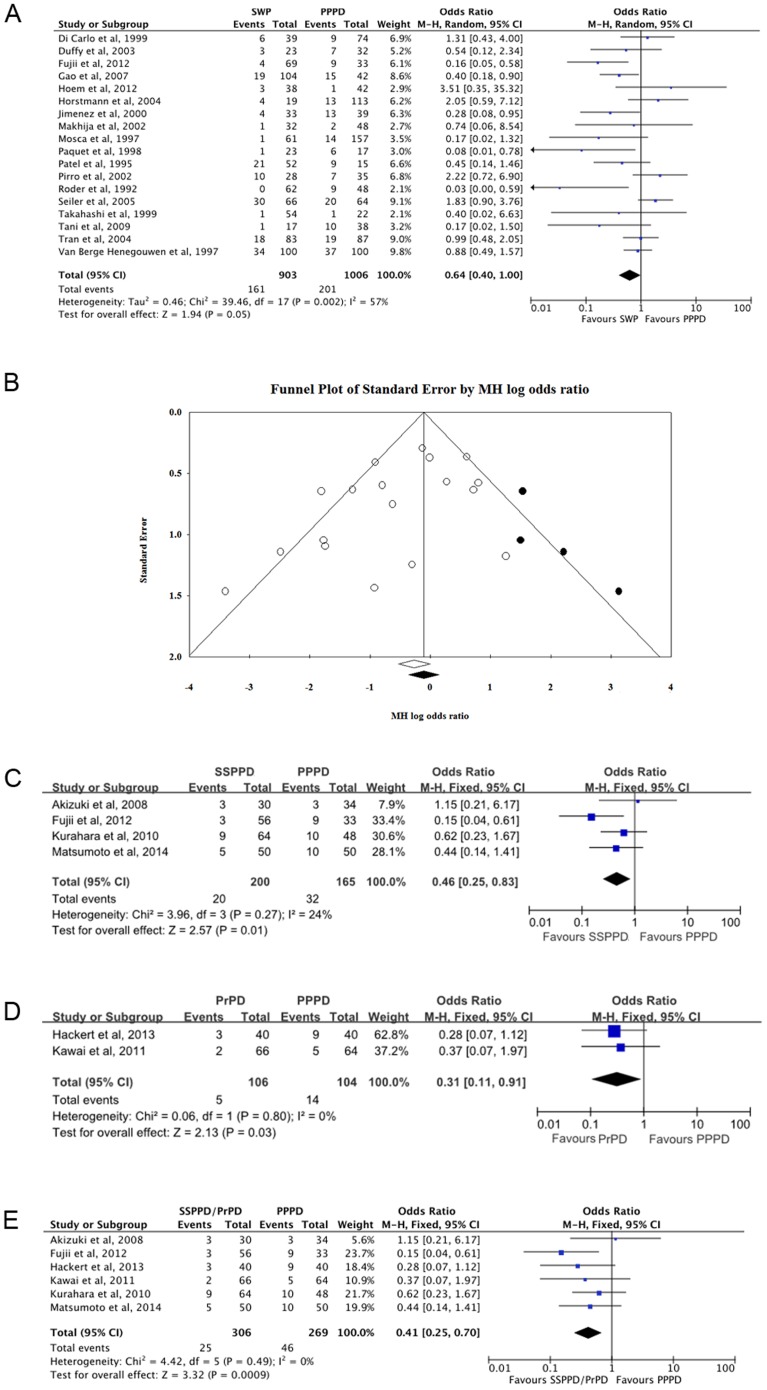
Subgroup analyses for delayed gastric emptying (DGE) incidence. **A**. Subgroup analysis of DGE for patients with SWP. **B**. Funnel plot of Duval's trim and fill correction of DGE incidence for the SWP studies. Included studies are illustrated as white dots. Potential missing studies are illustrate as black dots. **C**. Subgroup analysis of DGE for patients with SSPPD. **D**. Subgroup analysis of DGE for patients with PrPD. **E**. Subgroup analysis of DGE for patients with SSPPD/PrPD.

With respect to SWP, eighteen studies were included. The MH odds ratio was 0.64 (95% CI: 0.40–1.00; *P* = 0.05, random-effect model) with moderate heterogeneity (*I^2^* = 57%) (Figure 3A). Although publication bias was not significant by Egger's test (*P* = 0.069), Duval's trim and fill method could replace four missing studies to the right of the mean odds ratio (Figure 3B). After correction, the MH odds ratio was 0.85 (95% CI: 0.53–1.38).

#### The DGE incidence of SSPPD to PPPD was significantly different

With respect to SSPPD, four studies were included. The MH odds ratio was 0.46 (95% CI: 0.25–0.83; *P* = 0.01, fixed-effect model) with little heterogeneity (*I^2^* = 24%) (Figure 3C). No significant publication bias was detected by Egger's test (*P* = 0.56). The removal of the pylorus did reduce the incidence of DGE after SSPPD compared with PPPD.

#### The DGE incidence of PrPD to PPPD was significantly different

With respect to PrPD, only two studies were included. The MH odds ratio was 0.31 (95% CI: 0.11–0.91; *P* = 0.03, fixed-effect model) with little heterogeneity (*I^2^* = 0%) (Figure 3D). Egger's test was not conducted due to the low number of included studies. The removal of the pylorus did reduce the incidence of DGE after PrPD compared with PPPD.

#### The DGE incidence of SSPPD/PrPD to PPPD was significantly different

With respect to SSPPD/PrPD, six studies were included. The MH odds ratio was 0.41 (95% CI: 0.25–0.70; *P*<0.01, fixed-effect model) with little heterogeneity (*I^2^* = 0%) (Figure 3E). No significant publication bias was detected by Egger's test (*P* = 0.77). The removal of the pylorus did reduce the incidence of DGE after SSPPD/PrPD compared with PPPD.

### Secondary Outcomes

PRPD significantly increased blood loss (Figures S1), operation time (Figures S2) and mortality (Figures S4). Other parameters, including length of hospital stay (Figures S3), pancreatic fistula (Figures S5) and infection (Figures S6), were not significantly different between the PRPD and PPPD groups. Detailed information was summarized in Table 3.

**Table 3:** Outcomes of blood loss, operation time, and length of hospital stay, mortality, pancreatic fistula and infection after PRPD versus PPPD.

Outcomes	No. Patients	No. Studies	Risk Difference/Mean Difference (95% CI)	P value	*I^2^* index
Blood Loss	1417	16	273 (129, 418)	<0.01	67%
Operation Time	1408	16	35.6 (17.3, 53.9)	<0.01	75%
Length of Hospital Stay	1262	13	0.05 (−1.74, 1.85)	0.95	43%
Mortality	2487	26	0.02 (0.00, 0.03)	0.02	0%
Pancreatic Fistula	2467	26	0.00 (−0.03, 0.03)	0.96	41%
Infection	2229	24	0.01 (−0.01, 0.04)	0.29	0%

Abbreviations: PRPD, pylorus-removing pancreaticoduodenectomy; PPPD, pylorus-preserving pancreaticoduodenectomy.

## Discussion

DGE prolongs the hospitalization and the quality of life of patients. The removal of the pylorus is believed to influence the incidence of DGE, because postoperative pyloric dysfunction could occur secondary to devascularization and denervation [7], [8], [46]. Whether the removal of additional area of the pylorus during PPPD reduces the incidence of DGE has been investigated by several studies and is a matter of debate. It is necessary to realize that DGE is affected by various confounding factors, such as the route of reconstruction, prokinetic drug usage and the postoperative recovery program [3], [47], [48]. Different studies may not be consistent with respect to these factors, which would increase heterogeneity across studies. In contrast, a single study may suffer from an inadequate number of patients needed to reach adequate statistical power. Therefore, it is critical to identify the major confounding factors that would explain the heterogeneity. The subsequent analysis would be possible and meaningful after the exclusion of studies most accountable for the heterogeneity.

We first tested the heterogeneity concerning the incidence of DGE, which was moderate-high. To explore the potential variables, we conducted meta-regression analyses for the list of nineteen variables. Four of them showed statistical significance for the meta-regression. A longer solid food start time or NGT intubation time was associated with a greater reduction of DGE in the RPPD, compared with the PPPD group. In fact, the solid food start time was an important indicator of postoperative management. Balzano et al reported that the incidence of DGE would reduce for fast-track recovery program compared with the conventional method after pancreaticoduodenectomy [48]. Therefore, whether an additional resection of the pylorus could reduce the incidence of DGE should be evaluated within studies with a similar solid food start time and NGT intubation time. Another confounding factor, preoperative diabetes, has also been reported as a risk factor for DGE by a recent meta-analysis [15]. As for the number of patients in PPPD group, we sequentially excluded studies with the smallest patient number. When we excluded the studies with fewer than 15 patients, the patient number was no longer significantly contributed to the overall heterogeneity of DGE. In summary, four studies were excluded after meta-regression analysis, and a 12% reduction of *I^2^* index was achieved by the exclusion. A meta-analysis of the remaining 23 studies showed no significantly difference whether or not the pylorus was removed.

Next, we were interested in the different surgical techniques within the PRPD group. The major difference lay in the resected proportion of the stomach. The SSPPD and PrPD procedures only involve the resection of a relatively small portion of the pyloric stomach compared with SWP. SSPPD and PrPD are intended to preserve the pooling ability of the stomach [4]. Because DGE is closely related to stomach function, different surgical techniques in the PRPD group should be analyzed separately. A subgroup analysis showed that SSPPD, PrPD and SSPPD/PrPD, but not SWP, reduced the incidence of DGE. We interpreted the results in the following ways: 1) Preservation of the majority of stomach during the resection of the pylorus may help reduce the incidence of DGE; and 2) Because the SSPPD group only had four studies, PrPD group only contained only two studies, these results should be interpreted with cautions. More studies are needed to validate the results.

As for secondary outcomes, PRPD tended to involve a longer operation time, more blood loss and a higher mortality rate. However, the length of hospital stay was not significantly different. Similarly, the incidence of pancreatic fistula and infection was not significantly different between the two groups.

We acknowledged that our meta-analysis has several limitations. First, only four and two studies in SSPPD and PrPD group, respectively; therefore, the conclusion that removal of additional portions of the pylorus would significantly reduce the incidence of DGE after SSPPD or PrPD needs to be validated by more well-designed, prospective randomized studies with adequate patient sample sizes. Second, studies included in this meta-analysis were more non-RCTs than RCTs; therefore, cautions should be taken when interpreting the results from the analysis. We expect better designed studies to address whether or not SSPPD or PrPD will truly reduce DGE incidence.

In conclusion, this meta-analysis shows that pylorus removal doesn't reduce the overall incidence of DGE, compared with pylorus-preservation procedure. Subgroup analysis shows that pylorus removal may reduce the incidence of DGE when patients undergo SSPPD or PrPD. However, pylorus removal doesn't significantly reduce the incidence of DGE when patients undergo SWP.

## Supporting Information

Figure S1 **Forest plot of blood loss for included studies.** Blood loss is significant more in PRPD than PPPD. Abbreviations: PRPD, pylorus-removing pancreaticoduodenectomy; PPPD, pylorus-preserving pancreaticoduodenectomy.
(TIF)

Figure S2 **Forest plot of operation time for included studies.** Operation time is significant longer in PRPD than PPPD.Abbreviations: PRPD, pylorus-removing pancreaticoduodenectomy; PPPD, pylorus-preserving pancreaticoduodenectomy.
(TIF)

Figure S3 **Forest plot of length of hospital stay for included studies.** There is no significant difference between PRPD and PPPD.Abbreviations: PRPD, pylorus-removing pancreaticoduodenectomy; PPPD, pylorus-preserving pancreaticoduodenectomy.
(TIF)

Figure S4 **Forest plot of mortality for included studies.** Mortality incidence is significant higher in PRPD than PPPD.Abbreviations: PRPD, pylorus-removing pancreaticoduodenectomy; PPPD, pylorus-preserving pancreaticoduodenectomy.
(TIF)

Figure S5 **Forest plot of pancreatic fistula for included studies.** There is no significant difference between PRPD and PPPD.Abbreviations: PRPD, pylorus-removing pancreaticoduodenectomy; PPPD, pylorus-preserving pancreaticoduodenectomy.
(TIF)

Figure S6 **Forest plot of infection for included studies.** There is no significant difference between PRPD and PPPD.Abbreviations: PRPD, pylorus-removing pancreaticoduodenectomy; PPPD, pylorus-preserving pancreaticoduodenectomy.
(TIF)

Table S1 **Study quality assessment and the risk of publication bias by Newcastle-Ottawa quality tool for non-randomized studies.**
(DOCX)

Checklist S1 **PRISMA checklist for this meta-analysis.**
(DOC)
