# Supplementary figures and images for: The Effect of Pylorus Removal on Delayed Gastric Emptying after Pancreaticoduodenectomy: A Meta-Analysis of 2,599 Patients
Source: PLoS One. 2014 Oct 1;9(10):e108380. doi: 10.1371/journal.pone.0108380 (PMC4182728; doi:10.1371/journal.pone.0108380)

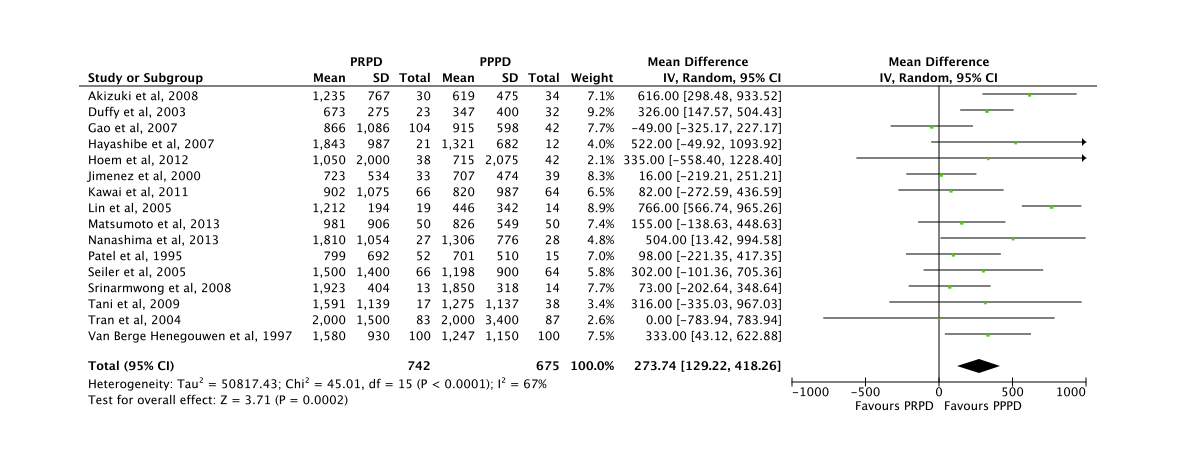

Supplement: Figure S1 — Forest plot of blood loss for included studies. Blood loss is significant more in PRPD than PPPD. Abbreviations: PRPD, pylorus-removing pancreaticoduodenectomy; PPPD, pylorus-preserving pancreaticoduodenectomy. (TIF) [file pone.0108380.s001.tif]

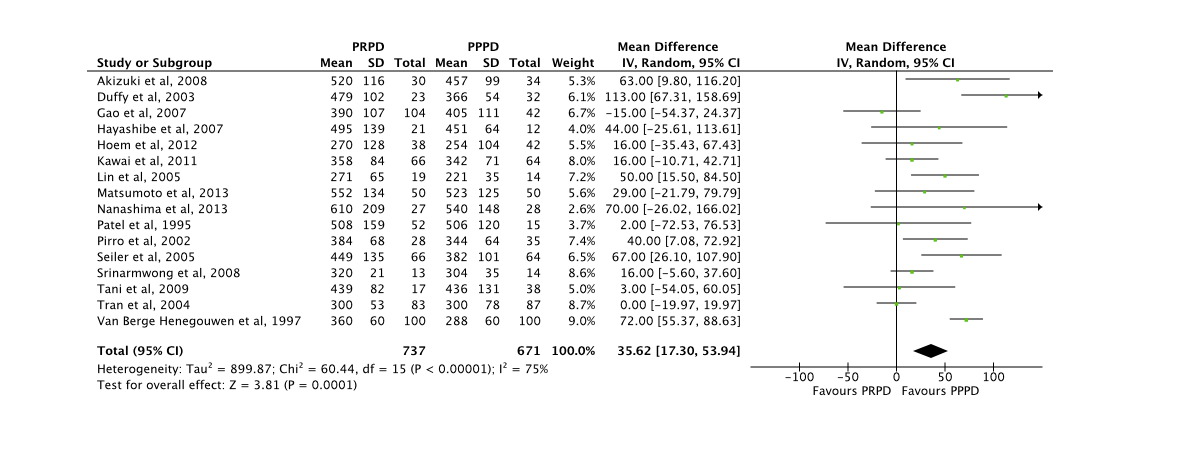

Supplement: Figure S2 — Forest plot of operation time for included studies. Operation time is significant longer in PRPD than PPPD.Abbreviations: PRPD, pylorus-removing pancreaticoduodenectomy; PPPD, pylorus-preserving pancreaticoduodenectomy. (TIF) [file pone.0108380.s002.tif]

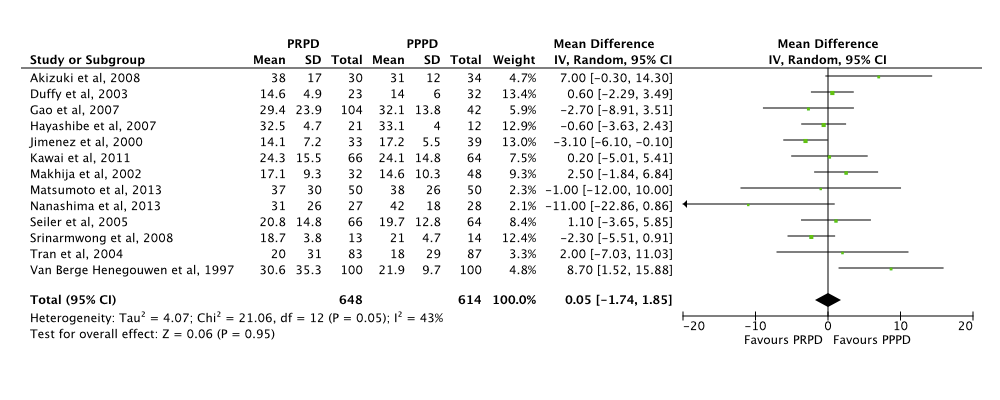

Supplement: Figure S3 — Forest plot of length of hospital stay for included studies. There is no significant difference between PRPD and PPPD.Abbreviations: PRPD, pylorus-removing pancreaticoduodenectomy; PPPD, pylorus-preserving pancreaticoduodenectomy. (TIF) [file pone.0108380.s003.tif]

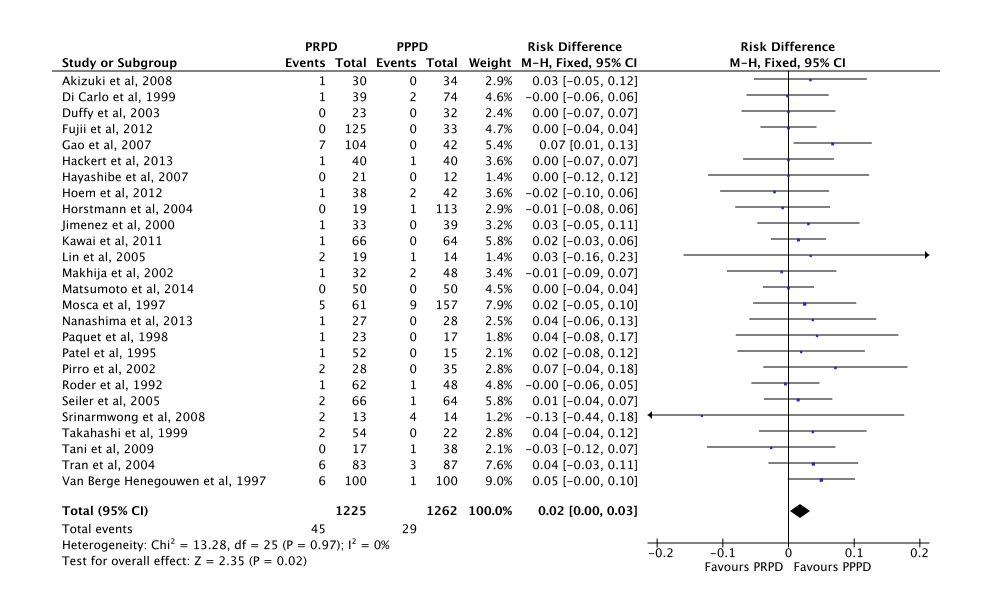

Supplement: Figure S4 — Forest plot of mortality for included studies. Mortality incidence is significant higher in PRPD than PPPD.Abbreviations: PRPD, pylorus-removing pancreaticoduodenectomy; PPPD, pylorus-preserving pancreaticoduodenectomy. (TIF) [file pone.0108380.s004.tif]

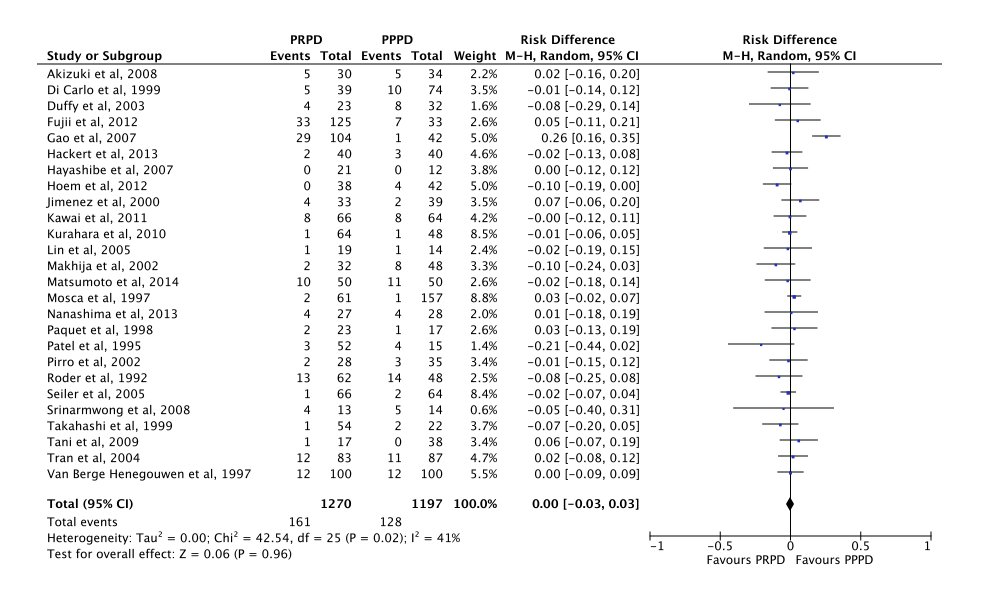

Supplement: Figure S5 — Forest plot of pancreatic fistula for included studies. There is no significant difference between PRPD and PPPD.Abbreviations: PRPD, pylorus-removing pancreaticoduodenectomy; PPPD, pylorus-preserving pancreaticoduodenectomy. (TIF) [file pone.0108380.s005.tif]

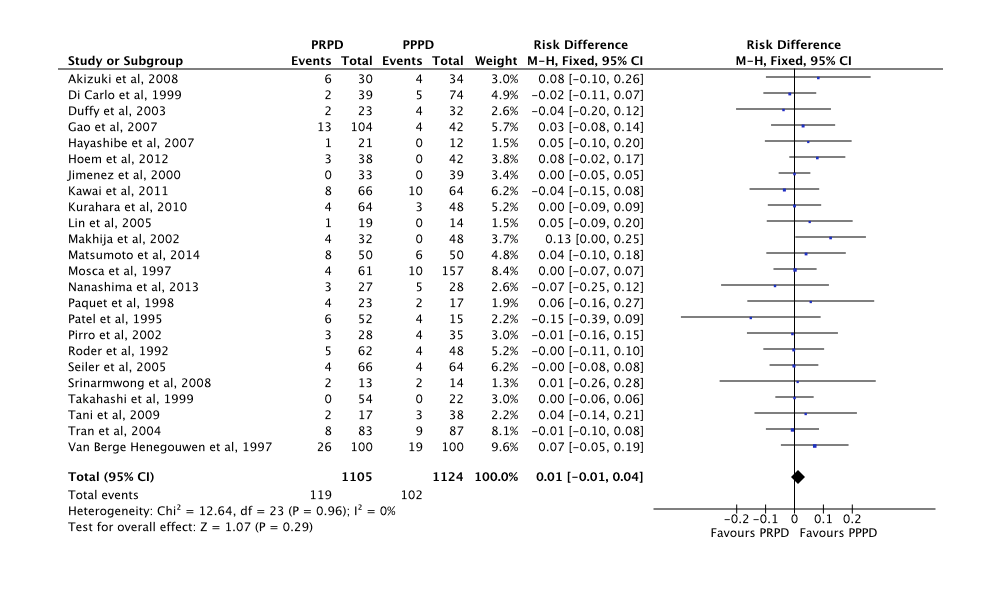

Supplement: Figure S6 — Forest plot of infection for included studies. There is no significant difference between PRPD and PPPD.Abbreviations: PRPD, pylorus-removing pancreaticoduodenectomy; PPPD, pylorus-preserving pancreaticoduodenectomy. (TIF) [file pone.0108380.s006.tif]
