# Supplementary material for: The Effect of Pylorus Removal on Delayed Gastric Emptying after Pancreaticoduodenectomy: A Meta-Analysis of 2,599 Patients
Source: PLoS One. 2014 Oct 1;9(10):e108380. doi: 10.1371/journal.pone.0108380 (PMC4182728; doi:10.1371/journal.pone.0108380)
Supplement: Table S1 — Study quality assessment and the risk of publication bias by Newcastle-Ottawa quality tool for non-randomized studies. (DOCX) [file pone.0108380.s007.docx]

Table S1: Study quality assessment and the risk of publication bias by Newcastle-Ottawa quality tool for non-randomized studies.

| Author | Year | Representativeness | Comparability | Exposure Ascertainment | Follow Up | Publication Bias |
| --- | --- | --- | --- | --- | --- | --- |
| Roder[[38](#_ENREF_38)] | 1992 | Same patient base | Age, gender, diagnosis were matched. All Patients were operated in the same hospital. | Surgical records | The follow-up was conducted prospectively to assess postoperative morbidity and survival. | Possible bias, due to nonrandomized allocation of patients into two groups |
| Van Berge Henegouwen[[44](#_ENREF_44)] | 1997 | Same patient base; consecutive enrolled | Age, gender, preoperative biliary drainage were matched. All Patients were operated in the same hospital. | Surgical records | The follow-up was conducted prospectively to assess postoperative morbidity. | Possible bias, due to nonrandomized allocation of patients into two groups |
| Di Carlo[[21](#_ENREF_21)] | 1999 | Same patient base | Age, gender, tumor stage and grade were matched. All patients were operated in the same hospital. | Surgical records | The follow-up was conducted to assess the survival of patients | Possible bias, due to nonrandomized allocation of patients into two groups |
| Makhija[[32](#_ENREF_32)] | 2002 | Same patient base | All Patients were operated in the same hospital. | Surgical records | The follow-up was conducted prospectively to assess postoperative morbidity | Possible bias, due to nonrandomized allocation of patients into groups. |
| Horstmann[[28](#_ENREF_28)] | 2004 | Same patient base; consecutive enrolled | Age, gender were matched. All Patients were operated in the same hospital. | Surgical records | The follow-up was conducted prospectively to assess postoperative morbidity | Possible bias, due to nonrandomized allocation of patients into two groups |
| Akizuki[[10](#_ENREF_10)] | 2008 | Same patient base; consecutive enrolled | Age, gender, diagnosis were matched. A single team of surgeons operated all patients. | Surgical records | The follow-up was conducted prospectively to assess postoperative morbidity | Possible bias, due to nonrandomized allocation of patients into two groups. |
| Hoem[[27](#_ENREF_27)] | 2012 | Same patient base | Age, gender, diagnosis were matched. | Surgical records | The follow-up was conducted prospectively to assess postoperative morbidity and survival | Possible bias, due to nonrandomized allocation of patients into two groups. |
| Hackert[[25](#_ENREF_25)] | 2013 | Same patient base | Age, gender, diagnosis leading to the resection were matched. All Patients were operated in the same hospital. | Surgical records | The follow-up was conducted prospectively to assess postoperative morbidity | Possible bias, due to nonrandomized allocation of patients into two groups. |
| Patel[[36](#_ENREF_36)] | 1995 | Same patient base | Age, gender, diagnosis were matched. All Patients were operated in the same hospital. | Surgical records | The follow-up was conducted retrospectively to assess postoperative morbidity and quality of life. | Possible bias, due to retrospective collected data and nonrandomized allocation of patients into two groups. |
| Mosca[[33](#_ENREF_33)] | 1997 | Same patient base | All Patients were operated in the same hospital. | Surgical records | The follow-up was conducted retrospectively to assess postoperative morbidity and survival. | Possible bias, due to retrospective collected data and nonrandomized allocation of patients into groups. |
| Takahashi[[41](#_ENREF_41)] | 1999 | Same patient base | All Patients were operated in the same hospital. | Surgical records | The follow-up was conducted retrospectively to assess postoperative morbidity and survival. | Possible bias, due to retrospective collected data and nonrandomized allocation of patients into two groups. |
| Jimenez[[29](#_ENREF_29)] | 2000 | Same patient base; consecutive enrolled | Age, gender, duration of pancreatitis and pain frequency were matched. All Patients were operated in the same hospital. | Surgical records | The follow-up was conducted retrospectively to assess postoperative morbidity and long-term nutritional status | Possible bias, due to retrospective collected data and nonrandomized allocation of patients into two groups. |
| Pirro[[37](#_ENREF_37)] | 2002 | Same patient base | All Patients were operated in the same hospital. | Surgical records | The follow-up was conducted retrospectively to assess postoperative morbidity and survival. | Possible bias, due to retrospective collected data and nonrandomized allocation of patients into two groups. |
| Duffy[[22](#_ENREF_22)] | 2003 | Same patient base; consecutive enrolled | Age, gender, race were matched. | Surgical records | The follow-up was conducted retrospectively to assess postoperative morbidity and survival | Possible bias, due to retrospective collected data and nonrandomized allocation of patients into two groups. |
| Gao[[24](#_ENREF_24)] | 2007 | Same patient base | Age, gender, diagnosis were matched. All Patients were operated in the same hospital. | Surgical records | The follow-up was conducted to assess postoperative morbidity | Possible bias, due to retrospective collected data and nonrandomized allocation of patients into two groups. |
| Hayashibe[[26](#_ENREF_26)] | 2007 | Same patient base | Age, gender, race, preoperative jaundice percentage and serum albumin were matched. All Patients were operated in the same hospital. | Surgical records | The follow-up was conducted to assess postoperative morbidity and nutritional status. | Possible bias, due to retrospective collected data and nonrandomized allocation of patients into two groups. |
| Tani[[42](#_ENREF_42)] | 2009 | Same patient base | Age, gender, preoperative biliary drainage were matched. All Patients were operated in the same hospital. | Surgical records | The follow-up was conducted retrospectively to assess postoperative morbidity and survival. | Possible bias, due to retrospective collected data and nonrandomized allocation of patients into two groups. |
| Kurahara[[30](#_ENREF_30)] | 2010 | Same patient base; consecutive enrolled | Age, gender, preoperative jaundice, diabetes mellitus status and previous abdominal surgery were matched. All Patients were operated in the same hospital. | Surgical records | The follow-up was conducted retrospectively to assess postoperative morbidity. | Possible bias, due to retrospective collected data and nonrandomized allocation of patients into two groups. |
| Fujii[[23](#_ENREF_23)] | 2012 | Same patient base; consecutive enrolled | Age, gender, preoperative body mass index, tumor histological type were matched; All Patients were operated in the same hospital. | Surgical records | The follow-up was conducted to assess postoperative morbidity, nutritional status and survival | Possible bias, due to retrospective collected data and nonrandomized allocation of patients into two groups. |
| Nanashima[[34](#_ENREF_34)] | 2013 | Same patient base | Age, gender, diagnosis were matched. All Patients were operated in the same hospital. | Surgical records | The follow-up was conducted retrospectively to assess postoperative morbidity. | Possible bias, due to retrospective collected data and nonrandomized allocation of patients into two groups. |
